# Th17 cytokines induce pro-fibrotic cytokines release from human eosinophils

**DOI:** 10.1186/1465-9921-14-34

**Published:** 2013-03-13

**Authors:** Saleh Al-Muhsen, Severine Letuve, Alejandro Vazquez-Tello, Mary Angeline Pureza, Hamdan Al-Jahdali, Ahmed S Bahammam, Qutayba Hamid, Rabih Halwani

**Affiliations:** 1 Asthma Research Chair and Prince Naif Center for Immunology Research, Department of Paediatrics, College of Medicine, King Saud University, Riyadh, Saudi Arabia; 2 Institut National de la Santé et de la Recherche Médicale (Inserm) U700 and Université Paris 7, Faculté de Médecine Denis Diderot, Site Bichat, Paris, France; 3 King Saud University for health sciences, Riyadh, Saudi Arabia; 4 Pulmonary Medicine Department, University Sleep Disorders Center, College of Medicine, King Saud University, Riyadh, Kingdom of Saudi Arabia; 5 Meakins-Christie Laboratories, McGill University, Montreal, QC, Canada

**Keywords:** Asthma, Eosinophils, Th17 cytokines, Pro-fibrotic cytokines, TGF-β, IL-11

## Abstract

**Background:**

Subepithelial fibrosis is one of the most critical structural changes affecting bronchial airway function during asthma. Eosinophils have been shown to contribute to the production of pro-fibrotic cytokines, TGF-β and IL-11, however, the mechanism regulating this process is not fully understood.

**Objective:**

In this report, we investigated whether cytokines associated with inflammation during asthma may induce eosinophils to produce pro-fibrotic cytokines.

**Methods:**

Eosinophils were isolated from peripheral blood of 10 asthmatics and 10 normal control subjects. Eosinophils were stimulated with Th1, Th2 and Th17 cytokines and the production of TGF-β and IL-11 was determined using real time PCR and ELISA assays.

**Results:**

The basal expression levels of eosinophil derived TGF-β and IL-11 cytokines were comparable between asthmatic and healthy individuals. Stimulating eosinophils with Th1 and Th2 cytokines did not induce expression of pro-fibrotic cytokines. However, stimulating eosinophils with Th17 cytokines resulted in the enhancement of TGF-β and IL-11 expression in asthmatic but not healthy individuals. This effect of IL-17 on eosinophils was dependent on p38 MAPK activation as inhibiting the phosphorylation of p38 MAPK, but not other kinases, inhibited IL-17 induced pro-fibrotic cytokine release.

**Conclusions:**

Th17 cytokines might contribute to airway fibrosis during asthma by enhancing production of eosinophil derived pro-fibrotic cytokines. Preventing the release of pro-fibrotic cytokines by blocking the effect of Th17 cytokines on eosinophils may prove to be beneficial in controlling fibrosis for disorders with IL-17 driven inflammation such as allergic and autoimmune diseases.

## Introduction

Asthma is a chronic inflammatory disorder of the lung that is usually associated with airway tissue remodelling. This term refers to the structural changes affecting lung tissue which normally include epithelial detachment, increased airway smooth muscle (ASM) mass, subepithelial fibrosis, mucous gland and goblet cell hyperplasia, vascular changes, and edema [1-4]. Subepithelial fibrosis is one of the most critical structural changes associated with airway remodeling. In normal subjects, a loose array of collagen fibrils resides beneath the basal membrane. In asthmatics, however, this layer is replaced by a dense network of extra-cellular matrix (ECM) proteins including collagens [5]. ECM protein deposition is known to be regulated by a number of cytokines and growth factors including TGF-β [6]. Several reports have shown that the majority of TGF-β1 mRNA positive cells in bronchial biopsies of severe asthmatics were eosinophils [7-9]. Eosinophils were also shown to produce IL-11 mRNA and protein [10]. These reports suggested that eosinophils could play an important role in regulating tissue fibrosis. IL-5 deficient mice experiments [11] and human studies [12] supported this hypothesis. In addition to lowering eosinophil levels, using anti-IL-5 antibodies was shown to be associated with reduced expression of ECM proteins particularly tenascin, lumican, and procollagen III [12].

Since its recent discovery, IL-17 has been described to be involved in various aspects of asthma pathogenesis. Elevated IL-17A levels were shown to correlate with increased airway hyper-responsiveness (AHR) in asthmatics [13]. In fact, IL-17 was shown to modulate airway structural cells leading to tissue remodeling. Over-expression of IL-17 F resulted in goblet cell hyperplasia and mucin gene expression [14]. In addition, using an in vitro cell migration assay, Change et al. have recently shown that Th17-associated cytokines IL-17A, IL-17 F, and IL-22 promote migration of human ASMCs. These effects were shown to be mediated by selective activation of receptors on ASMCs, with IL-17A and IL-17 F acting through p38 MAPK activation while IL-22 acting through a distinct nuclear factor kB (NF-kB)–dependent signaling pathway [15]. These studies indicated for a role of IL-17 in airway remodeling and hence in regulating asthma pathogenesis.

Eosinophils have receptors for a number of mediators that are associated with asthma including Th1, Th2, and Th17 cytokines [16-18]. The expression of IL-17 cytokines was also associated with subepithelial fibrosis [19-21]. In fact, Th17 cytokines were shown to trigger the expression of pro-fibrotic cytokines in bronchial fibroblasts [22]. We, hence, hypothesized that IL-17 cytokines may induce eosinophils to produce pro-fibrotic cytokines. In this paper, we stimulated eosinophils, isolated from normal and asthmatic subjects, with Th17 cytokines as well as a group of Th1 and Th2 cytokines known to be associated with asthma. Eosinophil production of TGF-β and IL-11 pro-fibrotic cytokines was then investigated.

## Materials and methods

### Study subjects

Ten subjects with severe asthma (6 males and 4 females, mean age 33.3 ± 2.6) who met the criteria defined by ATS on refractory asthma [23] were recruited. To be classified as severe asthmatics, patients must have had high-dose inhaled corticosteroid: Budesonide 160 μg/twice a day (or equivalent) or daily anti-leukotriene for >50% of the last year, and at least 1 other add-on therapy on daily basis for the previous 12 months. They were also required to have two of the following criteria: daily short-acting β-agonist, persistent FEV1 <60% and FEV1/FVC <75% predicted, 1 urgent visit or at least 3 steroid bursts in the previous year, prompt deterioration with <25% steroid dose reduction, or previous near-fatal asthma within the last 3 years. Subject characteristics are summarized in Table 1. Exclusion criteria included smoking history or any other pulmonary diseases or co-existing medical conditions such as cardiac and renal diseases and uncontrolled hypertension. Ten normal control subjects (6 males and 4 females, mean age: 38.2 ± 3.4) were also recruited. All normal control subjects were non-smokers with normal lung function, no history or symptoms of allergy and respiratory diseases, and were not taking any medications for the preceding four weeks. The Ethics Committee of the King Khalid University Hospital in Riyadh reviewed and approved the study, and all subjects recruited signed written informed consent for the drawing of peripheral venous blood for the isolation of eosinophils.

**Table 1:** Demography and spirometry data of the recruited subjects

	**Asthmatics**	**Healthy controls**
Age (years)	33.3 ± 2.6	38.2 ± 3.4
Males/females	6/4	6/4
Atopy (% of total)	100%	0
Duration of disease (years)	16.5 ± 9.8	n/a
Peripheral Blood Eosinophls	0.78 ± 0.04×10^9^/l	0.31 ± 0.02×10^9^/l
FEV1 (mean ± SD)	57.73 ± 2.56	107.5 ± 5.63
FVC (mean ± SD)	75.14 ±3.27	104.6 ± 6.87
FEV/FVC (mean ± SD)	66.34 ± 5.39	83 ± 4.62
Medications	- Inhaled corticosteroids: Symbicort II (Budesonide/Formoterol) (160/4.5 ug) (1–2 inhalations twice daily).	No
	- Anti-leukotriene: Singulair (montelukast sodium) (10 mg/d).	
	- Ventolin (albuterol) (as needed).	

Except for the medications indicated above, the patients were not receiving any other immunosupressive drugs. *FEV*_*1*_ forced expiratory volume; *FVC* forced vital capacity.

### Isolation and culture of eosinophils

Peripheral venous blood were drawn from patients with severe asthma (120-180 ml) (60 ml every 3 months) and from normal control subjects (180-240 ml) (60 ml every 3 months). Eosinophils were isolated by negative selection using MACS Isolation Kit (Miltenyi Biotec, Auburn, CA, USA) as previously described [24]. Neutrophils, monocytes and T cells were labeled with anti-CD16, anti-CD14 and anti-CD3 Abs respectively bound to immunemagnetic beads and separated with MACS LD Separation column. Eosinophil purity was consistently >98% as evaluated by Hema3 (Fisher) staining and the viability of freshly isolated eosinophils was >99% as evaluated by Trypan blue dye exclusion. Isolated eosinophils were then cultured in RPMI + 10% FCS in the presence of 30 pg/ml IL-5 cytokine required for eosinophil survival in vitro [25,26]. Eosinophil viability ranged between 85 and 92% following stimulation and culture.

### ELISA assay

Eosinophils (2×10^6^ cells/ml cultured in 24 well plate) were stimulated with Th1 (IL-2, IFN-γ) (50 ng/ml), Th2 (IL-4, IL-5, IL-9, IL-13) (50 ng/ml), and Th17 (IL-17A, IL-17 F, IL-23) (10, 25, 50, or 100 ng/ml) cytokines (R&D Systems, Minneapolis, Minn., USA) for 24 hrs and supernatants were collected. In some experiments, eosinophils were treated with p38 mitogen-activated protein kinase (MAPK) inhibitors (SB 203580; 5 μM, Invivogen San Diego, CA, USA) or PI3K inhibitor (PI103; 5 μM, Cayman Chemical, Ann Arbor, Mich., USA) 2 hours prior to stimulation with IL-17. Levels of secreted TGF-β and IL-11 in supernatants were determined using ELISA assay (R&D Systems, Minneapolis, Minn., USA) according to the manufacturer instructions.

### RNA extraction and real-time RT-PCR

Eosinophils were stimulated with cytokines (Th1 (50 ng/ml), Th2 (50 ng/ml) or Th17 (50 ng/ml)) for 4 hours prior to cell harvest. In some experiments, eosinophils were treated with p38 MAPK inhibitors (SB 203580; 5 μM), or PI3K inhibitor (PI103; 5 μM) 2 hours prior to stimulation with IL-17. Cells were then harvested, total RNA extracted (levels of RNA extracted from 2×10^6^ eosinophils were 18.4 ± 4.7 μg for asthmatic eosinophils and 16.3 ± 3.9 μg for healthy controls) (RNeasy Mini kit, Qiagen, CA, USA) and modulations of the level of expression of TGF-β and IL-11 mRNA were determined using quantitative RT-PCR (Applied Biosystems, 7900 Fast RT-PCR system). Specific primers for TGF-β and IL-11 were as follows: TGF-β: Forward: 5-CTGGACACCCTAACCGTGAT-3, Reverse: 5-CTAGGCCGTGCTGCTGCT-3; IL-11: Forward: 5-GTGGCCAGATACAGCTGTCGC-3, Reverse: 5- GGTAGGACAGTAGGTCCGCTC-3. Relative expressions of TGF-β and IL-11 genes normalized with GAPDH were determined by the delta-delta Ct method [27].

### Assessment of p38 MAPK phosphorylation by western analysis

2×10^6^ eosinophil cells were starved using medium with 0.1% FBS for 18 hours. Cells were stimulated with 50 ng/mL IL-17A and IL-17 F for 0, 10, and 20 minutes and total proteins were extracted using lysis buffer (1% Triton X-100 containing protease and phosphatase inhibitor cocktails (Roche, Mannheim, Germany). Protein lysates (10 μg) were then resolved on 10% acrylamide SDS-PAGE gel and blots were probed with antibodies to phosphorylated p38 MAPK (Millipore) and total p38 MAPK (Millipore). Membranes were analyzed with an Odyssey IR scanner using Odyssey imaging software 3.0 (LI-COR Biosciences, Inc).

### Statistical analysis

Data are presented as mean ± SD. Expression of pro-fibrotic cytokines was evaluated using ANOVA followed by Bonferroni-Dunn *post hoc* test. Non-parametric Mann–Whitney *U* test (Systat, version 7.0, SPSS, Chicago, IL) was used to evaluate significance in differential phosphorylation of MAPK. Values of p < 0.05 were considered statistically significant.

## Results

### Th1 and Th2 cytokines do not induce expression of eosinophil derived pro-fibrotic cytokines

**Figure 1:**
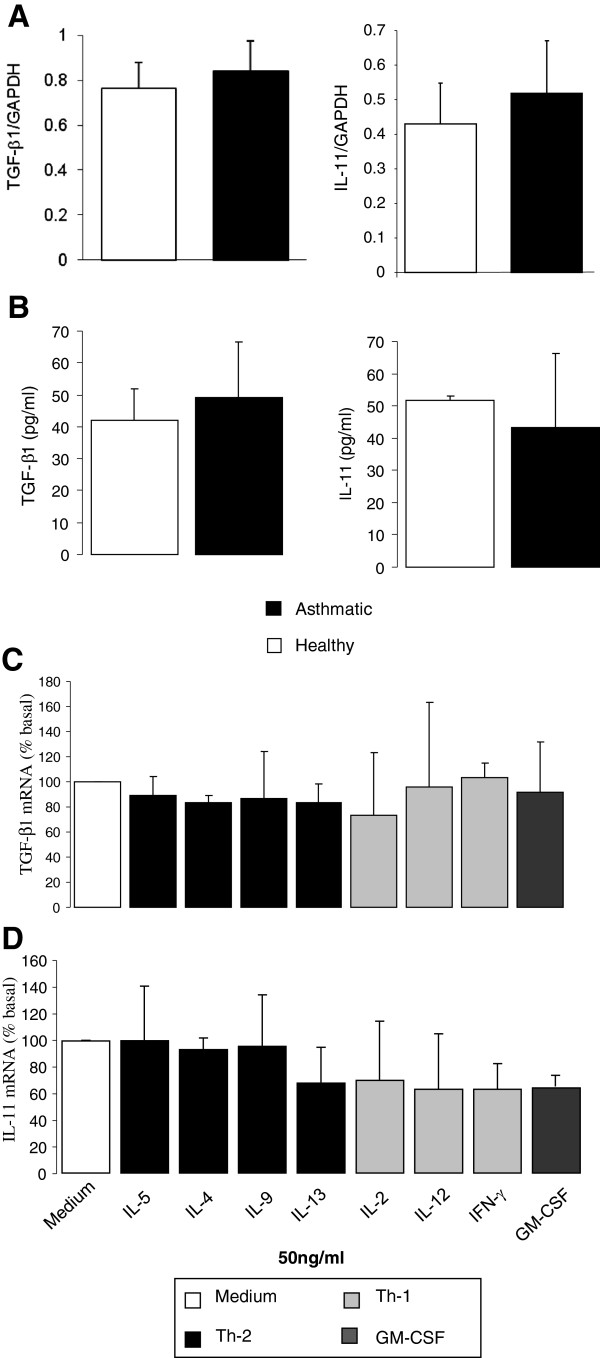
**Basal expression of pro-fibrotic cytokines by human eosinophils isolated from asthmatic and controls subjects.** Eosinophils were isolated from 10 asthmatic and 10 controls subjects and total RNA was extracted from 2×10^6^ million cells and quantified using real-time PCR. **A**: Level of expression of TGF-β1 and IL-11 mRNA in eosinophils of asthmatic versus control subjects (n = 10). **B**: Levels of TGF-β1 and IL-11 cytokines within the supernatant of un-stimulated eosinophils (n = 10) as determined by ELISA assay. (**C-D**) Effect of Th1 and Th2 cytokines on asthmatic eosinophil TGF-β1 and IL-11 transcripts levels. Level of expression of TGF-β1 (**C**) and IL-11 (**D**) mRNA as quantified by real-time PCR following 4 hours exposure to mediators. Data is presented as percentage of basal expression (n = 10).

The patho-physiological characteristics of lung tissue inflammation during severe asthma differ significantly from those of the milder disease. While the airway tissues of mild asthmatics usually present preferential Th2 cytokine profile [28], those from severe asthmatics show a Th17 lymphocyte infiltration and elevated cytokine levels, particularly Th1 cytokines (IFN-γ, IL-2), IL-17 and TGF-β [29-31]. Many T-helper cytokines were shown to play a significant role in regulating TGF-β expression and function in different types of cells [32-34]. However, their direct role in regulating eosinophil ability to produce pro-fibrotic cytokines was not studied. To investigate that, we first determined the basal expression levels of pro-fibrotic cytokines within peripheral blood eosinophils of 10 asthmatic and non-asthmatic individuals using real time RT-PCR. The levels of expression of TGF-β and IL-11 mRNA in eosinophils isolated from asthmatic individuals (ct values: TGF-β: 27.53 ± 0.21 IL-11: 28.80 ± 1.2) were comparable to those isolated from healthy controls (ct values: TGF-β: 27.70 ± 0.29 IL-11: 29.56 ± 0.86) (Figure 1A). Eosinophil supernatant IL-11 and TGF-β cytokines levels were also determined in the two groups using ELISA assay (Figure 1B). Similarly, no change in the secreted levels of these pro-fibrotic cytokines was detected between the two groups. We then investigated whether Th1 and Th2 cytokines play a role in regulating eosinophils pro-fibrotic cytokines production. To do that, we stimulated 2×10^6^ eosinophil cells isolated from 10 asthmatic as well as healthy individuals with Th1 (IL-2, IL-12, and IFN-γ), and Th2 (IL-4, IL-5, IL-9, and IL-13) cytokines as well as GM-CSF for 4 hrs. Total RNA was then extracted from stimulated eosinophils and the level of IL-11 and TGF-β was determined using real time RT-PCR. As shown in Figure 1C-D, stimulating asthmatic eosinophils with Th1 or Th2 cytokines did not affect TGF-β (ct values range: 27.63 ± 0.21 to 27.58 ± 0.79) (Figure 1C) or IL-11 (ct values range: 28.67 ± 0.84 to 28.76 ± 0.18) (Figure 1D) m-RNA levels. Similar results were obtained at higher concentrations of Th1 and Th2 cytokines (100 ng/ml) as well as for eosinophils isolated from healthy controls (data not shown). These results indicated that neither Th1 nor Th2 cytokines play a significant role in regulating expression of eosinophil derived pro-fibrotic cytokines.

### Th17 cytokines enhance the expression of eosinophil derived pro-fibrotic cytokines in asthmatic individuals

**Figure 2:**
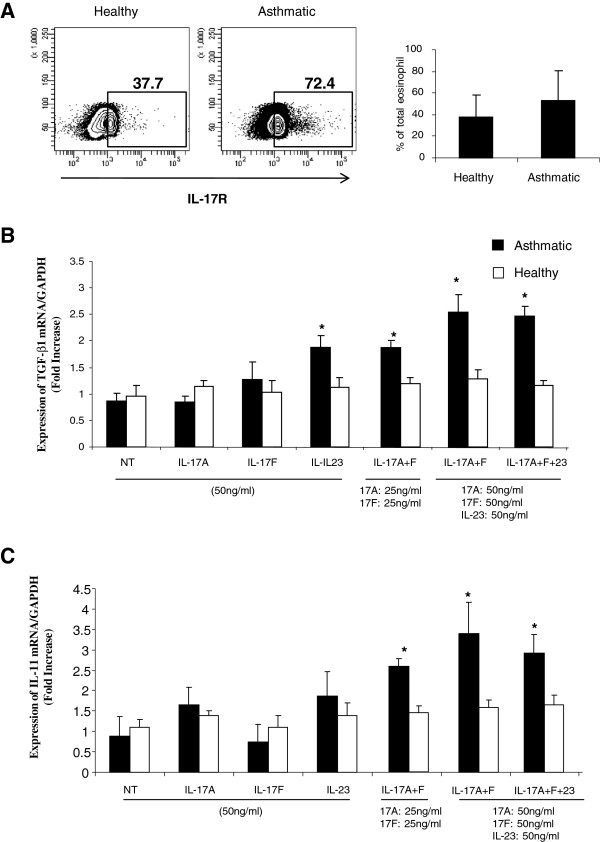
**IL-17 and IL-23 enhance eosinophil expression of pro-fibrotic cytokines.** (**A**) Surface expression of IL-17R on eosinophils (1×10^6^ cells) isolated from healthy and asthmatics was determined by flow cytometry. Blots are representative data for eosinophils isolated from one healthy control and one asthmatic patient. The graph shows arithmetic mean ± SD of IL-17R positive eosinophils as percentage of total eosinophils (n = 5). 2×10^6^ peripheral blood eosinophils isolated from 10 asthmatic and 10 controls subjects were stimulated with IL-17A, F, and IL-23 (50 ng/ml or 25 ng/ml) alone or in combination for 4 hrs. Total RNA was extracted and mRNA levels of TGF-β and IL-11 were then quantified using real-time PCR. mRNA expression levels of TGF-β (**B**) and IL-11 (**C**) were normalized with GAPDH for asthmatic versus healthy individuals.

**Figure 3:**
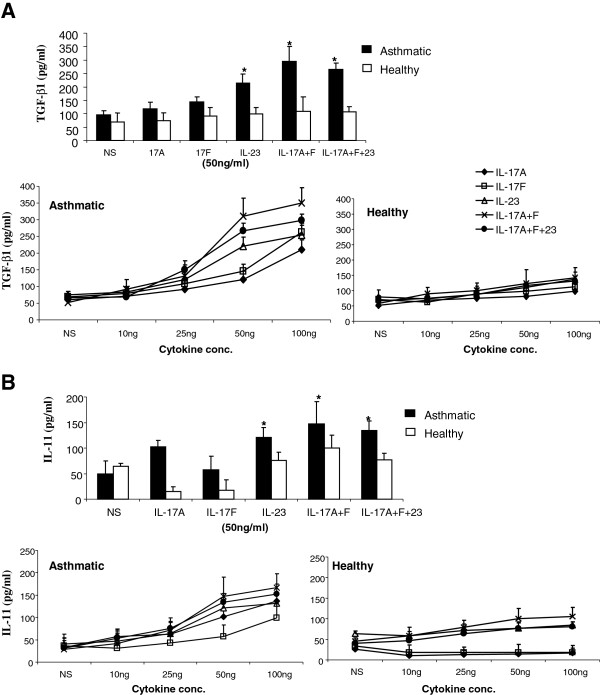
**Th17 cytokines enhance eosinophil production and release of pro-fibrotic cytokines.** Levels of TGF-β (**A**) and IL-11 (**B**) in the supernatant of stimulated eosinophils (1×10^6^ cells/0.5 ml) were determined 24 hrs following Th17 cytokine stimulation (0-100 ng/ml) using ELISA assay. Results are expressed as the arithmetic mean ± SD from 5 independent experiments. * = p < 0.05.

IL-17A enhanced the production of IL-6 and IL-11 in bronchial fibroblasts [22] while IL-17 F was shown to induce the expression of TGF-β in human umbilical vein endothelial cells (HUVECs) [35]. IL-17A and IL-17 F were recently shown to be over expressed in bronchial lung tissue of asthmatic patients compared to healthy controls [29] and their level of expression was associated with the severity of the diseases. Interestingly, using FACS and western analysis, eosinophils were also shown to express receptors for Th17 cytokines [16]. We, therefore, hypothesised that Th17 cytokines may induce eosinophils to produce pro-fibrotic cytokines. To investigate that, we first determined the expression levels of IL-17R on eosinophils isolated from both groups. As indicated in Figure 2A, eosinophils from both healthy and asthmatic subjects express IL-17R. Although asthmatic eosinophils express higher levels of IL-17R, this increase did not reach significance. We next stimulated 2×10^6^ eosinophils, isolated from 10 severe asthmatic patients and 10 healthy controls, with IL-17A, IL17F, as well as IL-23, another Th17 cytokine for 4 hrs. Total RNA was then extracted and eosinophil expression of TGF-β and IL-11 mRNA was measured using real-time PCR. As shown in Figure 2B, contrary to stimulating eosinophils with IL-17A and IL-17 F alone, stimulation with a combination of IL-17A + F (ct value: 24.39 ± 0.17, P = 0.031; n = 10), or IL-23 (ct value: 24.42 ± 0.21, P = 0.025; n = 10) alone, induced a significant increase in the expression of eosinophil derived TGF-β. Further increase in TGF-β expression was observed when stimulating with double the amount of the combined cytokines IL-17A + F (ct value: 24.06 ± 0.4, P = 0.012; n = 10) and or IL-17A + F + IL-23 (ct values: 23.98 ± 0.16, P = 0.018; n = 10). Interestingly, this increase in TGF-β production was only observed within eosinophils isolated from asthmatic patients. Stimulation of eosinophils isolated from non-asthmatic individuals with Th17 cytokines had no effect on TGF-β production (ct values: IL-17A + F: 25.29 ± 0.15; IL-23: 25.22 ± 0.18; IL-17A + F (50 ng): 25.36 ± 0.14; IL-17A + F + 23 (50 ng): 25.78 ± 0.11, p = NS) (Figure 2B). Similarly, a combination of IL-17A and IL-17 F at different concentrations or IL-17A + F + IL-23 induced a significant increase in IL-11 mRNA expression within eosinophils isolated from asthmatics (ct values: IL-17A + F (25 ng): 24.61 ± 0.37, p = 0.029; IL-17A + F (50 ng): 23.71 ± 0.70, p = 0.009; IL-17A + F + 23 (50 ng): 23.80 ± 0.37, p = 0.014, n = 10) but not healthy subjects (ct values: IL-17A + F (25 ng): 25.18 ± 0.10; IL-17A + F (50 ng): 25.10 ± 0.11; IL-17A + F + 23 (50 ng): 25.00 ± 0.13, p = NS) (Figure 2C). To determine effective concentration inducing eosinophils release of TGF-β and IL-11 cytokines, a dose response effect of Th17 cytokines was performed. Eosinophils were treated with increasing concentration of Th17 cytokines and levels of TGF-β and IL-11 in their supernatant were determined using ELISA assay (Figure 3A). Although low concentrations of Th17 cytokines induced pro-fibrotic cytokine secretion, a significant enhancement of TGF-β and IL-11 release was only attained at 50 ng/ml and above. At this concentration, the level of eosinophil derived TGF-β was significantly increased following treatment with a combination of IL-17A + F (P = 0.032; n = 10), IL-23 (P = 0.049; n = 10) alone, or IL-17A + F + IL-23 (P = 0.043; n = 10) (Figure 3A). Similarly, IL-11 secreted levels were significantly upregulated following stimulation with a combination of IL-17A + F (P = 0.035; n = 10), IL-23 (P = 0.048; n = 10) alone, or IL-17A + F + IL-23 (P = 0.043; n = 10) (Figure 3B). This data suggest that, in an asthmatic environment, an additive effect of Th17 cytokines enhance the production of eosinophils derived pro-fibrotic cytokines.

### IL-17 cytokine enhance eosinophil derived TGF-β and IL-11 production through P38 MAP kinase activation

**Figure 4:**
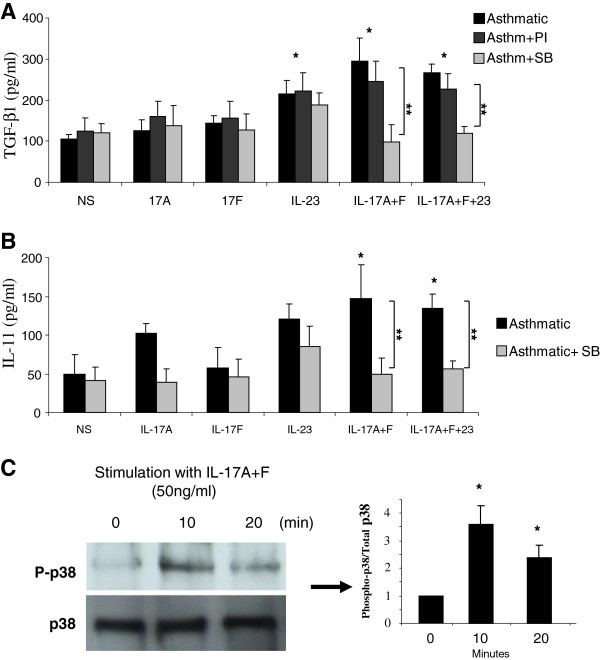
**P38 MAP Kinase activation is required for IL-17 enhancement of eosinophil derived pro-fibrotic cytokines.** Eosinophils were isolated from peripheral blood of 10 asthmatic patients and 2×10^6^/ml cells were treated, or not, with p38 MAPK or PI3K inhibitors (SB2035802 and PI103, respectively) 2 hours prior to stimulation with IL-17 (50 ng/ml). Levels of TGF-β (**A**) and IL-11 (**B**) in the supernatant of stimulated eosinophils were then determined 24 hrs following Th17 cytokine stimulation using ELISA assay (n = 10). (**C**) Induction of p38 MAPK phosphorylation by a combination of IL-17A and IL-17 F (50 ng/ml each) is detected by western analysis. The western data shown represent one of similar results from 4 independent experiments. * = p < 0.05 compared to non-stimulated (NS). ** = p < 0.05 compared to stimulated not inhibited.

P38 mitogen-activated protein kinase (MAPK), being at a critical junction of the IL-17 signaling pathways, has been shown by various reports to be a key regulator element for the activity of IL-17 cytokines [15,16,36]. To study the mechanism behind Th17 cytokines enhancement of eosinophil derived TGF-β production, eosinophils were isolated from peripheral blood of 10 asthmatic patients as described above. 2×10^6^ cells were treated, or not, with p38 MAPK or PI3K inhibitors (SB2035802 and PI103, respectively), or diluent control (DMSO) 2 hours prior to stimulation with IL-17. As shown in Figure 4, inhibiting phosphorylation of p38 MAPK significantly decreased the level of TGF-β (P = 0.011 (IL-17A + F); P = 0.015 (IL-17A + F + 23); n = 10) and IL-11 (P = 0.021 (IL-17A + F); P = 0.026 (IL-17A + F + 23); n = 10) secreted into eosinophil supernatants 24 hrs following Th17 cytokine stimulation (Figure 4A, B). This blocking effect was only specific to p38 MAPK as diluent control or inhibitor of another kinase (PI3K) did not affect the supernatant levels of TGF-β and IL-11 (P = NS; n = 10). This data indicated that p38 MAPK activation is critical for IL-17 induced eosinophil derived pro-fibrotic cytokine production. To confirm p38 MAPK phosphorylation following treatment with IL-17 cytokines, 2×10^6^ eosinophil cell were treated with IL-17A + F (50 ng/ml each) for 0, 10 and 20 minutes and the level of p38 MAPK phosphorylation was then determined using western analysis. As shown in Figure 4C, stimulating eosinophils with a combination of IL-17A and IL-17 F resulted in phosphorylation of p38 MAPK which seems to peak at 10 minutes (3.5 fold increase, p = 0.039). Inhibiting p38 MAPK, PI3K, or ERK1/2, however, did not interfere with the ability of IL-23 to stimulate eosinophil to produce pro-fibrotic cytokines. This indicated that IL-23 may use other mechanisms to stimulate pro-fibrotic cytokine release that need to be further investigated.

## Discussion

Eosinophils constitute a major source of TGF-β in asthmatic lung tissue [7-9]. Reduction of lung eosinophilia by anti–IL-5 therapy in humans [12] or genetic knock down in mice [11] significantly reduced airway fibrosis and pulmonary TGF-β1 levels. Here, we show, for the first time, that Th17 cytokines enhance eosinophil derived TGF-β and IL-11 production. This effect of Th17 cytokines was prominent on eosinophils isolated from asthmatics but not healthy subjects. Our results clearly demonstrate that eosinophils constitute an additional site of action for Th17 cytokines in asthma supporting a role for IL-17 in regulating fibrosis and airway remodeling.

Although Th2 (IL-4, IL-5, and IL-13) cytokines has earlier been reported to regulate the expression of TGF-β1 by eosinophils [37,38], other studies had shown no effect of these cytokines on TGF-β expression [39]. Our results support the latest reports as we did not see any increase in TGF-β or IL-11 mRNA or protein expression following stimulation with Th2 cytokines. Similarly, Th1 cytokines had no effect on eosinophil derived TGF-β expression. In fact, IFN-γ was previously shown to inhibit TGF-β production in human airway epithelial cells which is in consistence with our findings [38].

The enhancement of eosinophil derived pro-fibrotic cytokine release upon IL-17 cytokines stimulation was only significant in eosinophils isolated from asthmatic individuals. Although there was a slight upregulation of TGF-β and IL-11 expression in eosinophils isolated from healthy individuals upon IL-17 stimulation, this increase did not reach significance. Peripheral blood eosinophils of asthmatic patients were shown to be primed compared to those of healthy subjects [40-42] which may render them more susceptible to IL-17 effect. Our results suggest that IL-17 cytokines enhance pro-fibrotic activity of activated, such as in the case of allergic and auto-immune diseases, but not resting eosinophils. Furthermore, our data indicated that asthmatic eosinophils may express higher levels of IL-17R than those of healthy controls (Figure 2A). IL-23 was shown to increase expression of IL-17RA and IL-17RC in eosinophils [16] and hence this observed potential increase in IL-17R in asthmatic eosinophils could be due to increased serum IL-23 in those patients. Serum levels of IL-23 were shown to inversely correlate with level of pulmonary function (FEV1) of asthmatic patients in various reports [43,44]. This may indicate that, due to the expected increase in serum IL-23 with asthma severity, eosinophils isolated from mild and moderate asthmatic patients may express higher levels of IL-17 receptors than eosinophils of healthy controls but lower than those of severe asthmatic patients. Understanding the correlation between asthmatic patients’ IL-23 serum levels, the expression of IL-17R on peripheral blood eosinophils, and the severity of asthma requires further investigations.

Eosinophils are known to produce IL-17 cytokines [22] and IL-23 was shown to stimulate the expression of IL-17A cytokine [45]. This may indicate that IL-23 could stimulate eosinophils release of pro-fibrotic cytokines indirectly by triggering their release of IL-17A. This possibility, however, needs to be further investigated.

Stimulating eosinophils with IL-17 cytokines at a physiologically relevant concentration (25 ng/ml) resulted in an increase in TGF-β and IL-11 production although not to a significant levels (Figure 3). While stimulating eosinophils with either IL-17A or F alone did not enhance a significant increase in pro-fibrotic cytokines, using a combination of both cytokines did indicating an additive effect. Since both IL-17A and IL-17 F share the same IL-17R receptor [46], a concentration of around 25 ng/ml or more of each IL-17 cytokine seems to be required for efficient eosinophil derived pro-fibrotic cytokine release. This is more likely to be achieved in vivo through the additive effect of IL-17A and F rather than a high concentration of a single IL-17 cytokine alone.

Accumulating evidences from various reports indicate for a key role of p38 MAPK pathway in IL-17 cytokine activity on structural and inflammatory cells in asthma [15,36]. Binding of IL-17A and F to the IL-17RA and RC receptors on target cells triggers the recruitment of the U-box E3 ubiquitin ligase Act1 (CIKS). Act1 will in turn recruit TGF-β activated kinase that serves as the template for the activation of the transcription factors NF-kB, CEBPb (beta), as well as the MAPK pathways ERK1/ERK2 and p38 MAPK [47]. P38 MAPK, ERK, and JNK pathways were shown to regulate TGF-β transcription each in response to different stimuli [48]. Our data suggest that IL-17 cytokines stimulate TGF-β transcription via the activation of p38 MAPK but not PI3K or ERK1/2 MAPK (data not shown) pathways. IL-23, however, seems to use another mechanism as inhibiting those pathways did not affect its ability to stimulate TGF-β and IL-11 production.

## Conclusions

Data presented herein suggest a new role for Th17 cytokines in airway remodeling during asthma. IL-17 cytokines seem to contribute to airway tissue fibrosis by enhancing production of eosinophil derived pro-fibrotic cytokines. This role of IL-17 was dependent on p38 MAPK activation. Therefore, upstream activators of p38 MAPK within the IL-17R pathway may represent an attractive target in corticosteroid-unresponsive diseases [49,50]. Preventing the release of TGF-β by blocking the effect of IL-17 on eosinophils may also prove efficient in controlling fibrosis for disorders with IL-17 driven inflammation such as allergic and autoimmune diseases.

## Competing interests

The authors declare that they have no competing interests.

## Authors’ contributions

SM carried out the real-time PCR for Th17 experiments and participated in the design of the study. SL carried out the real-time PCR and ELISA for Th1 and Th2 experiments. AVT participated in the design of the study and performed the statistical analysis. MAP carried out the ELISA for Th17 experiments. HJ contributed in recruiting patients to the study. QH contributed in study design and data analysis. RH conceived of the study, lead efforts on its design and coordination and finalized the manuscript draft. All authors read and approved the final manuscript.
